# Early Diagnosis of Classic Homocystinuria in Kuwait through Newborn Screening: A 6-Year Experience

**DOI:** 10.3390/ijns7030056

**Published:** 2021-08-17

**Authors:** Hind Alsharhan, Amir A. Ahmed, Naser M. Ali, Ahmad Alahmad, Buthaina Albash, Reem M. Elshafie, Sumaya Alkanderi, Usama M. Elkazzaz, Parakkal Xavier Cyril, Rehab M. Abdelrahman, Alaa A. Elmonairy, Samia M. Ibrahim, Yasser M. E. Elfeky, Doaa I. Sadik, Sara D. Al-Enezi, Ayman M. Salloum, Yadav Girish, Mohammad Al-Ali, Dina G. Ramadan, Rasha Alsafi, May Al-Rushood, Laila Bastaki

**Affiliations:** 1 Department of Pediatrics, Faculty of Medicine, Kuwait University, P.O. Box 24923, Safat 13110, Kuwait; 2 Department of Pediatrics, Farwaniya Hospital, Ministry of Health, Sabah Al-Nasser 92426, Kuwait; 3 Kuwait Medical Genetics Center, Ministry of Health, Sulaibikhat 80901, Kuwait; buthina@hotmail.com (B.A.); a.ar92@yahoo.com (R.M.E.); sumayaalkanderi@gmail.com (S.A.); alaagenetic@hotmail.com (A.A.E.); doaadoaaibrahim@yahoo.com (D.I.S.); lailabastaki16@yahoo.com (L.B.); 4 Newborn Screening Laboratory, Kuwait Medical Genetics Center, Ministry of Health, Sulaibikhat 80901, Kuwait; amiral6666@gmail.com (A.A.A.); rehabm1998@yahoo.com (R.M.A.); biochemmay@gmail.com (M.A.-R.); 5 Next Generation Sequencing Laboratory, Kuwait Medical Genetics Center, Ministry of Health, Sulaibikhat 80901, Kuwait; drnasermali@gmail.com (N.M.A.); dralali@kmgc.gov.kw (M.A.-A.); 6 Molecular Genetics Laboratory, Kuwait Medical Genetics Center, Ministry of Health, Sulaibikhat 80901, Kuwait; ahmadalahmad@moh.gov.kw (A.A.); a.z.n.al-enezi@live.com (S.D.A.-E.); 7 Newborn Screening Office, Farwaniya Hospital, Ministry of Health, Sabah Al-Nasser 92426, Kuwait; usama.kazaz100@gmail.com; 8 Newborn Screening Office, Adan Hospital, Ministry of Health, Hadiya 52700, Kuwait; dr.cyrilxavier@gmail.com; 9 Newborn Screening Office, Al-Sabah Maternity Hospital, Ministry of Health, Sulaibikhat 80901, Kuwait; semsemamarch@yahoo.com; 10 Newborn Screening Office, Jahra Hospital, Ministry of Health, Jahra 00020, Kuwait; yasserelfeky3@gmail.com; 11 Biochemistry Laboratory, Al-Sabah Hospital, Ministry of Health, Shuwaikh 70051, Kuwait; draymacha@gmail.com; 12 Clinical Biochemistry Laboratory, Ibn Sina Hospital, Ministry of Health, Shuwaikh, P.O. Box 25427, Safat 13115, Kuwait; drgirish@yahoo.com; 13 Department of Pediatrics, Al-Sabah Hospital, Ministry of Health, Shuweikh 70051, Kuwait; dinaramadan@yahoo.com; 14 Department of Pediatrics, Adan Hospital, Ministry of Health, Hadiya 52700, Kuwait; ralsafi@moh.gov.kw

**Keywords:** classic homocystinuria, methionine, molecular testing, newborn screening, total homocysteine, incidence

## Abstract

Kuwait is a small Arabian Gulf country with a high rate of consanguinity and where a national newborn screening program was expanded in October 2014 to include a wide range of endocrine and metabolic disorders. A retrospective study conducted between January 2015 and December 2020 revealed a total of 304,086 newborns have been screened in Kuwait. Six newborns were diagnosed with classic homocystinuria with an incidence of 1:50,000, which is not as high as in Qatar but higher than the global incidence. Molecular testing for five of them has revealed three previously reported pathogenic variants in the *CBS* gene, c.969G>A, p.(Trp323Ter); c.982G>A, p.(Asp328Asn); and the Qatari founder variant c.1006C>T, p.(Arg336Cys). This is the first study to review the screening of newborns in Kuwait for classic homocystinuria, starting with the detection of elevated blood methionine and providing a follow-up strategy for positive results, including plasma total homocysteine and amino acid analyses. Further, we have demonstrated an increase in the specificity of the current newborn screening test for classic homocystinuria by including the methionine to phenylalanine ratio along with the elevated methionine blood levels in first-tier testing. Here, we provide evidence that the newborn screening in Kuwait has led to the early detection of classic homocystinuria cases and enabled the affected individuals to lead active and productive lives.

## 1. Introduction

Kuwait is a small country situated in the northwestern part of the Arabian Gulf with a total area of 17,818 square kilometers [1]. As of 2021, Kuwait has a population of 4.5 million; 1.3 million are Kuwaitis and 3.2 million are expatriates mainly from India, Egypt, Bangladesh, the Philippines, and other Asian and Arabian countries [2]. There are six main governmental hospitals where individuals with inborn errors of metabolism (IEM) are managed. Like other Arabian Gulf and other Arab countries, a high rate of consanguinity has been observed in Kuwait, with a reported rate that exceeds 50% [3], resulting in the high prevalence of autosomal recessive disorders [4].

**Figure 1:**
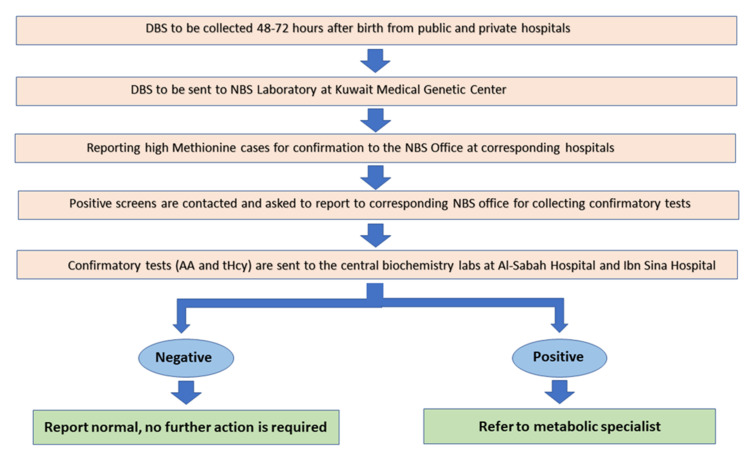
The process of the newborn screening for classic homocystinuria in Kuwait. DBS: dried blood spot; NBS: newborn screening; AA: plasma amino acids; tHcy: total homocysteine.

In October 2014, the Kuwait Ministry of Health started a publicly funded expanded newborn screening program (NBS) meeting the highest international standards to screen for a wide range of metabolic and endocrine disorders, including a total of 22 disorders (Table 1) via testing dried blood spots (DBS) and thus replacing the old, limited NBS for congenital hypothyroidism and phenylketonuria that was introduced in 2005. The NBS program aims to screen all infants born in Kuwait; it initially only covered public hospitals until April 2015 when the private hospitals were included as they used to perform NBS testing individually. In May 2019, the NBS program, centered at the NBS Laboratory in the Kuwait Medical Genetics Center (KMGC), started covering 100% of the neonates born in Kuwait (Table 2; Figure 1).

Classical homocystinuria (HCU) (OMIM 236200), is an inborn error of methionine (Met) and homocysteine (Hcy) metabolism with a global incidence of ~1:260,000 [5], and the highest incidence of pyridoxine-nonresponsive HCU reported in Qatar, affecting 1 in 1800 [6,7,8]. However, the true frequency is still unknown and is thought to be higher than the incidence detected by NBS [9,10]. HCU is an autosomal recessive disorder, caused by deficiency of the cystathionine beta synthase (CBS) enzyme, pyridoxine-dependent, resulting in an elevated blood, urine, and tissue levels of Hcy and its precursor Met in blood, urine, and tissues. HCU is the most common inborn error of sulfur metabolism [11].

The clinical manifestations of untreated HCU include cognitive impairment, behavioral problems, ocular abnormalities (ectopia lentis, myopia), connective tissue involvement (marfanoid habitus, osteoporosis), and thromboembolism [12]. Due to the favorable outcome of patients treated early with diet, betaine, and/or pyridoxine, NBS for HCU is recommended [13,14]. HCU can be screened in DBS by determining Met, methionine-to-phenylalanine (Met/Phe) ratio, and total homocysteine (tHcy) as a second-tier marker [15].

The management of HCU resides mainly in lowering Hcy level to near normal levels via Met restricted diet, betaine, folic acid, and pyridoxine supplementation. However, poor compliance to diet and medications as well as the considerable fraction of pyridoxine nonresponsive individuals noted in the Qatari population [6], has led to further therapies that are currently under investigations, such as enzyme replacement and gene therapies [16].

This paper is the first to review the current practice of the national NBS for HCU in the State of Kuwait as well as the follow-up strategy for positive screens since the expansion of the NBS program in October 2014.

## 2. Materials and Methods

### 2.1. NBS Registry

A retrospective analysis of the data registry for the NBS over the 6-year period between January 2015 and December 2020 in Kuwait was performed after obtaining consent from the NBS program and KMGC. These data included newborns delivered at both private and public hospitals all over Kuwait according to the percentage of coverage shown in Table 2. Data on metabolite concentrations in DBS at the time of screening obtained from all newborns were reviewed and only DBS detecting hypermethioninemia were included in this study.

### 2.2. DBS Collection Protocol

The first DBS samples were collected on Whatman 903 filter papers within 48–72 h of life but could be accepted up to one month after birth. For any DBS collected before 48 h of age, a second DBS would be repeated within 7 days. All DBS were sent to the NBS laboratory at the KMGC for analysis. Samples showing high Met levels were typically repeated three times from different punches from the same DBS filter paper to ensure that the DBS was homogenous with adequate quality for accurate quantitative analysis. The mean value of the three readings of Met was reported as positive initial screen if it exceeded the cutoff value. Positive initial screens were followed by a second DBS for amino acid analysis in addition to the plasma total homocysteine (tHcy) analysis at Al-Sabah Hospital Biochemistry Laboratory and Ibn Sina Hospital Biochemistry Laboratory respectively, the main laboratories that provide diagnostic testing for metabolic disorders, as recommended in NBS ACT Sheets and Confirmatory Algorithms for Met by the American College of Medical Genetics to confirm or exclude the diagnosis of HCU [17] (Figure 1). If the second DBS continued to show an elevated Met, quantitative plasma amino acid analysis would then be performed on a new sample.

### 2.3. Analytical Methods

#### 2.3.1. First DBS

The method used to measure Met in the first DBS is semi-quantitative determination via tandem mass spectrometry (MS/MS) without a butylation step [18,19]. Electrospray ionization tandem mass spectrometry (ESI-MS/MS) analysis was performed using a Waters Triple Quadrupole Mass Spectrometer (Xevo TQD from Waters manufacture). The analytical measurements were performed in multiple reaction monitoring mode (MRM) using NeoLynx software. The stable isotope amino acid and acylcarnitine internal standards, supplied by Chromsystems, measures 11 amino acids, free carnitine, and 30 different acylcarnitines. To monitor the performance of our assays, quality control (QC) was run with the same sample plate.

#### 2.3.2. Amino Acids in Repeat (Second) DBS:

An API 3200 triple quadrupole tandem mass spectrometer (AB-SCIEX) and liquid chromatography-tandem mass spectrometry (LC–MS/MS) were used to analyze amino acids with the butylation step, as previously described with minor modifications [20,21].

#### 2.3.3. Plasma Amino Acids

High-performance liquid chromatography (HPLC) (Sykam S 433 Amino Acid Analyzer system) was used to analyze plasma amino acids as previously described with minor modifications [22].

#### 2.3.4. Plasma Total Homocysteine

Plasma total homocysteine was measured by competitive immunoassay as previously described [23,24].

#### 2.3.5. Evaluation of Met/Phe Ratio

Phe was analyzed in DBS similarly to Met using MS/MS. We have measured the Met/Phe ratio for all NBS samples (338,379) for the period 2015–2020, including the six confirmed HCU cases. We have then compared the Met/Phe ratio to Met levels as a primary marker for HCU and determined its specificity as a screening test. The Met/Phe ratios ranged from 0.2 to 3.4 with an average of 0.3 and standard deviation of 0.09. The Met/Phe cutoff of 0.75 corresponds to the 99th percentile of the controls. All statistical analyses were performed using SPSS22.

### 2.4. Molecular Testing

All biochemically confirmed HCU cases, except for the first individual (Table 3), underwent clinical genetic testing by either targeted variant testing using polymerase chain reaction (PCR) amplification followed by Sanger sequencing, or through next generation sequencing technology using Ion AmpliSeq Inborn Errors of Metabolism community panel (ThermoFisher Scientific, Waltham, MA, USA). To identify potential disease-causing variants, we focused on homozygous or compound heterozygous variants in *CBS* gene due to their association with HCU. Variants were further prioritized if they were rare in gnomAD database (less than 1.0% population allele frequency) https://gnomad.broadinstitute.org/ (accessed on 27 May 2021) or if they were previously reported to be associated with HCU in the literature.

## 3. Results

### 3.1. NBS Registry

The Kuwait NBS data registry included 338,379 samples for 304,086 screened neonates (171,218 = 56% Kuwaiti; 132,868 = 44% non-Kuwaiti) born in Kuwait between January 2015 and December 2020 (Table 2). The number of screened samples are typically higher than the number of screened newborns since the protocol of national NBS of Kuwait recommends collecting three DBS for premature babies over the first month of life. Further, DBS that are collected before 48 h are required to have a second DBS collected within the first week of life.

### 3.2. DBS with Hypermethioninemia

About 400 screened newborns, including both preterm and full-term babies, had hypermethioninemia with an initial cutoff value of 50 μmol/L (corresponding to the 99.9 percentile) for the period between 2015 and 2017, which was decreased to 44 μmol/L (mean+ 5SD) thereafter. If we apply the cutoff of Met > 44 μmol/L (mean + 5SD) for the period 2015 until 2020, the total number of newborns with exclusive hypermethioninemia is 512. A total of six confirmed cases of HCU based on elevated plasma Met and tHcy have been identified since the year 2015 through the NBS program. These six cases (4 male/2 female) were from six different families, four of whom were Kuwaitis, one was Saudi Arabian, and the last individual was Egyptian (Table 3). This has resulted in an overall incidence of 1:50,000 and of 1:43,000 among only Kuwaiti newborns. All cases were pyridoxine-nonresponsive and had high Met levels > 50 μmol/L with Met/Phe > 1 and elevated tHcy (except P1 which tHcy level was unavailable). The final NBS result was available at a median age of 4 days (range 3–5 days) and a specific treatment plan was initiated at a median age of 9 days (range 5–16 days) (Table 3). Molecular testing of five affected newborns identified three homozygous variants in *CBS* gene: c.969G>A, p.(Trp323Ter); c.1006C>T, p.(Arg336Cys); and c.982G>A, p.(Asp328Asn) (Table 3). 

### 3.3. Evaluation of Met/Phe Ratio as a Potential Strategy in Screening for HCU in Kuwait

Using a Met/Phe cutoff of 0.75 in addition to Met levels (with a cutoff of 44 μmol/L), led to a percentage of 0.05 (174 cases) of suspected HCU (specificity 0.999, Table 4), which does not significantly differ from using Met > 44 μmol/L only as a primary marker. However, it has increased the positive predictive value (PPV) of HCU screening from 1.17% to 3.4% (Table 4). Applying Met/Phe ratio with different cutoffs (0.65, 0.70, 0.75, 0.80, 0.85) have resulted in a slight increase in the specificity of HCU screening, respectively, as shown in Table 4.

## 4. Discussion

Our report is the first to describe the expanded NBS program in Kuwait since its launch in October 2014. 

In the current study, we report the outcome of the NBS program for HCU in Kuwait between January 2015 and December 2020. The biochemical hallmarks of CBS deficiency include highly elevated concentrations of plasma tHcy combined with low plasma cystine or total cysteine, elevated plasma Met, low normal to decreased cystathionine, and grossly abnormal cystathionine/Met ratio [15,25,26]. The NBS for HCU has been based on the detection of increased concentrations of Met in DBS, which is a nonspecific marker and has poor diagnostic sensitivity for HCU [27,28,29,30,31,32] as it might be influenced by the time of screening (false negative rate is more likely seen with earlier sampling) [29]. Further, breastfed newborns would have lower Met blood levels compared to formula-fed babies [29,33]. Prematurity, low birth weight, parental nutrition (TPN) and liver disease are additional causes for secondary hypermethioninemia. Further causes of hypermethioninemia include IEM of Met, S-adenosylmethionine and S-adenosylhomocysteine, such as deficiencies of Met adenosyltransferase I and III (MAT I/III), glycine N-methyltransferase (GNMT), and s-adenosylhomocysteine (AdoHcy) hydrolase, and citrin deficiency [14,27,34]. Further, deficiency of fumarylacetoacetate hydrolase (tyrosinemia type 1) could be associated with secondary hypermethioninemia due to the associated liver damage and/or accumulation of fumarylacetoacetate, thus inhibiting MAT [27]. Most individuals with pyridoxine-responsive HCU are typically missed with the current NBS strategies based on detecting high Met [15,27]. Missed cases of pyridoxine-nonresponsive HCU (about 20–50%) were reported when high Met cutoff values were used [7,35]; lowering the Met cutoff from 138 to 67 μmol/L has doubled the detection rate of CBS deficiency and having the Met cutoff values as low as 40 μmol/L has increased the NBS sensitivity in the United States [31,32,36]. Met/Phe ratio has been used in HCU screening as Phe metabolism is not affected by the disease, and only abnormal Met values would be detectable such that overall higher or lower amino acid profiles would have normal ratios [29]. Per Okun et al., using plasma Met > 40 μmol/L as first-tier would result in about 40% of samples to be measured for tHcy in a second-tier strategy compared to using Met/Phe ratio at 0.56 as a first-tier strategy, which would result in about 10% of samples to be analyzed for tHcy resulting in 100% sensitivity and specificity in NBS for HCU [29]. Therefore, setting a low cutoff value for Met and including Met/Phe ratio are two main factors to increase the sensitivity of the NBS for HCU [36]. The measurement of tHcy in DBS using MS/MS has been shown to be reliable [36,37,38] and is being used as a first-tier NBS marker only in Qatar [7,14,32,36,39]. Per Keller et al., the implementation of tHcy for all homocystinurias and methylmalonic acid for the combined remethylation disorders, as second-tier markers, would increase the specificity of the NBS, but have not yet been widely applied in NBS practice [14].

In Kuwait, the NBS is based on detection high Met (> 44 μmol/L) in DBS followed by collecting another blood specimen for biochemical confirmatory tests for positive cases, which includes analyzing blood for tHcy and amino acid chromatography. The positive rate based on the first Met DBS screening was about 1:846 (0.12%) (400/338,379) (Table 2). Based on our study, we have noticed a high rate of hypermethioninemia in the first DBS screening; there were 66 false positive cases for each one true positive case (400/6) which is considered as high false positive rate with low PPV of about 1.5% (Table 2). Therefore, implementing the Met/Phe ratio in the first tier along with the blood Met levels could be considered to decrease the false positive rate and increase the PPV. Indeed, we have applied the Met/Phe ratio with a cutoff 0.75 as a second marker along with Met cutoff > 44 μmol/L (Table 3,Table 4), which has resulted in an increase in the specificity and PPV in HCU screening (Table 3, Table 4) compared to using the blood Met levels or Met/Phe ratio as the only marker (Table 4). 

As demonstrated by Al-Dewik et al. and Yamada et al., individuals with late diagnoses of HCU have a poorer outcome, mainly in terms of social and intellectual outcomes compared to individuals diagnosed based on NBS and treated early in infancy, emphasizing the importance of early diagnosis of treatment via NBS [40,41]. However, despite early diagnosis of HCU by NBS and dietary and pharmacological compliance, affected individuals continued to develop some of the disease complications [40]. In this study, the final NBS result was available at a median age of 4 days, and specific treatment was initiated at a median age of 9 days (Table 3). Further studies of the natural history of all individuals diagnosed with HCU is important to demonstrate the clinical outcome of those diagnosed early via NBS compared to individuals with late diagnoses.

The incidence of HCU in Kuwait in this study is shown to be 1:50,000, relatively similar to the incidence reported in Saudi Arabia [42]. With the high rate of consanguinity in Kuwait and the rest of Middle East countries, where consanguinity can reach over 50% [3,43,44,45], it may be surprising to have a low incidence of HCU in Kuwait compared to the neighboring Arabian Gulf country of Qatar, where the highest global incidence of HCU has been reported [6]. This could be attributed to the lack of confirmation of true positive screens due to failure in claiming a second recall samples, failure to obtain a repeat DBS for those who have been collected before 48 hours of age, or false negative result of the first DBS, which is less likely given the low Met cutoff value used. Further, Kuwait population is heterogenous, composed of different neighboring ethnicities with the majority being expatriates, which could be another reason for the low incidence of HCU compared to the native Qatari population, which is more homogenous and populated by original Qatari tribes and, thus, genetically isolated [7,46]. Nonetheless, the incidence of HCU in Kuwait is relatively high compared to the other parts of the world, where the incidence of HCU reaches 1:260,000 worldwide, 1:222,000 in Latin America [5], 1:1,120,000 in Japan, 1:492,000 in Korea, 1:132,000 in Germany [47], which emphasizes the importance and effectiveness of NBS in our consanguineous population with the increased incidence of autosomal recessive disorders in general and metabolic disorders specifically. 

In Kuwait, the biochemical confirmation of HCU is typically followed by a confirmatory molecular testing for the CBS gene. Molecular testing of affected newborns as well as late diagnosed individuals with HCU has revealed the same founder missense variant reported in Qatar, c.1006C>T, p.(Arg336Cys) [6], as well as two other previously reported pathogenic variants, one reported in Saudi population, c.969G>A, p.(Yrp323Ter) [48], while the other variant c.982G>A, p.(Asp328Asn), was previously reported in two affected individuals of Filipino and Indian ethnicities [49,50] (Table 3).

Our experience of NBS for HCU in Kuwait is successful for several reasons: 1. The high consanguinity rate in the population and thus the high incidence of IEM; 2. The small size of the country that allowed centralization of NBS laboratory screening service and facilitated the close coordination between different hospitals including private sectors; 3. NBS and early treatment not only reduce the morbidity and mortality typically associated with IEM but also allow for carrier screening of at-risk family members via molecular testing, enabling a sort of prevention strategy, providing better counselling options for the families, and reducing the future incidence of the disease.

## 5. Conclusions

In summary, our study is the first to review the experience of the NBS program for HCU in Kuwait. We demonstrated that our national NBS for HCU is highly effective, the screened positive cases are successfully followed up, and the affected infants are treated within an average of 9 days of their lives. We report the incidence of HCU in Kuwait to be 1 in 50,000 since the expansion of the NBS program in October 2014, emphasizing its effectiveness and importance. Further, we recommend adding Met/Phe ratio to blood Met level as a first-tier and tHcy as a second-tier strategy in our national HCU screening to further increase the specificity of NBS testing.

## Figures and Tables

**Table 1:** List of endocrine and metabolic disorders as well as hearing and pulse oximetry screen integrated into the national newborn screening program in Kuwait. CHD: congenital heart defects.

Group	Disorder
Endocrinopathies	Congenital hypothyroidism
	Congenital adrenal hyperplasia
Aminoacidopathies	Argininosuccinic aciduria (ASA lyase deficiency)
	Citrullinemia (ASA synthetase deficiency)
	Homocystinuria (cystathionine synthase def.)
	Maple syrup urine disease (MSUD)
	Phenylketonuria (PKU)
	Tyrosinemia (Type I)
Fatty Acid Oxidation Disorders	Long chain hydroxy acyl-CoA dehydrogenase deficiency (LCHAD)
	Medium chain acyl-CoA dehydrogenase def. (MCAD)
	Trifunctional protein deficiency (TFP)
	Very long chain acyl-CoA dehydrogenase deficiency (VLCAD)
Organic Acidemias	3-Methylcrotonyl-CoA carboxylase deficiency (3MCC)
	3-Hydroxy-3-methylglutaryl-CoA lyase deficiency (3HMG-CoA lyase deficiency)
	Beta ketothiolase deficiency (mitochondrial acetoacetyl CoA thiolase deficiency)
	Glutaric acidemia type I (GA-I))
	Isovaleric acidemia (IVA)
	Methyl malonic acidemia (MMA)
	Multiple CoA carboxylase deficiency (MCD)
	Propionic acidemia (PA)
Galactosemia	Classic galactosemia
Biotinidase Deficiency	
Hearing Loss	
Pulse Oximetry for CHD	

**Table 2:** Overview of births in Kuwait and samples screened through the national newborn screening program over the 6-year period between January 2015 and December 2020.

	2020	2019	2018	2017	2016	2015	Total
Total samples received in NBS laboratory	56,441	56,333	55,210	59,655	57,951	52,789	338,379
Total newborns screened in Kuwait	51,315	50,916	48,501	53,689	52,155	47,510	304,086
Total premature newborns screened ≤ 33 wks	2823	3263	3312	3350	3495	3298	19,541
Newborns ≤ 33 wks (with exclusive high methionine)	35	28	43	46	16	12	180
Newborns >33 wks (with exclusive high methionine)	49	17	38	73	19	24	220
No. of all newborns in Kuwait per CSB	NA	53,565	56,121	59,172	58,797	59,271	NA
No. of Kuwaiti newborns per CSB	NA	32,263	33,168	33,680	33,431	33,581	NA
No. of Non-Kuwaiti newborns per CSB	NA	21,302	22,953	25,492	25,366	25,690	NA
Screened Kuwaiti newborns	29,762 *	30,145	28,645	29,074	28,733	24,859	171,218
Screened non-Kuwaiti newborns	21,553 *	20,771	19,856	24,615	23,422	22,651	132,868
Newborns not screened under national NBS program	0 ^ψ^	2649	7620	5483	6642	11,761	34,155
Percent of coverage of national NBS program (%)	100 ^ψ^	94.8	84.3	89.8	87.3	75.2	88.8

CSB: central statistical bureau; NA: not available at central statistical bureau (CSB) website; No.: number; wks: weeks. * The newborns’ nationalities for the year 2020 are based on the maternal nationalities as CSB data are not yet available for that year. ^ψ^ Assuming that all newborns were covered since CSB data for the year 2020 is unavailable yet.

**Table 3:** Overview of individuals diagnosed with classic homocystinuria via newborn screening program in the period 2015–2020 in Kuwait, demonstrating the nationality, sex, date of birth, gestational age, time between birth and start of treatment, pathogenic variants detected in *CBS* gene, and biochemical results.

	Nationality	Sex	Date of Birth (Month. Year)	Gestational Age (Weeks)	Age at NBS Result (Days)	Age at Start of Treatment (Days)	* DNA Variant	Protein Variant	Zygosity	1st DBS Met Levels (Cutoff 44 μmol/L)	Met/Phe (Cutoff 0.75)	tHcy Levels (Cutoff 15 μmol/L)
** P1 **	KSA	M	07.2015	39	5	NA	NA	NA	NA	52	NA	NA
** P2 **	K	M	01.2017	38	4	16	c.969G>A	Trp323Ter	Homo	94.2	1.5	98
** P3 **	K	F	05.2017	39	3	9	c.969G>A	Trp323Ter	Homo	75	1.37	147
** P4 **	E	F	03.2019	37	4	5	c.982G>A	Asp328Asn	Homo	119	1.59	132
** P5 **	K	M	05.2019	40	5	12	c.1006C>T	Arg336Cys	Homo	63.2	1.02	113
** P6 **	K	M	01.2020	37	4	7	c.969G>A	Trp323Ter	Homo	93.28	1.9	111

DBS: dried blood spots; E: Egyptian; Homo: homozygous; K: Kuwaiti; KSA: Kingdom of Saudi Arabia; Met: methionine; NA: not available; NBS: newborn screening; P: patient; Phe: phenylalanine; tHcy: total blood homocysteine. * The reference transcript is NM_001178008.2 (hg19/GRCh3

**Table 4:** Results of applying Met/Phe ratio to the current NBS strategy of measuring Met in DBS as first-tier with cutoffs trimmed for 100% sensitivity for all newborn samples in the period 2015–2020.

Cutoff	Sensitivity	Specificity	No. (%) of Positives	PPV %
Met > 44 μmol/L	1	0.998	512 ^э^ (0.15%)	1.17
Met > 44 μmol/L& Met/Phe > 0.75	1	0.999	174 (0.05%)	3.4
Met/Phe > 0.65	1	0.996	1190 (0.35%)	0.5
Met/Phe > 0.70	1	0.998	658 (0.19)	0.9
Met/Phe > 0.75 ^ψ^	1	0.998	417 (0.12%)	1.4
Met/Phe > 0.80	1	0.999	286 (0.08%)	2.1
Met/Phe > 0.85	1	0.999	206 (0.06%)	2.9

Met: methionine; No.: number; Phe: phenylalanine; PPV: positive predictive value. ^ψ^ Cutoff (Met/Phe ratio average + 5 × SD) = 0.75. ^э^ Calculating total newborns (full-term and preterm) with Met cutoff value > 44 μmol/L for the period 2015–2020. Of note, the initial Met cutoff was 50 μmol/L for the period 2015 to 2017, then was decreased to 44 μmol/L thereafter.

## References

[1] Kuwait Government Online Geography of Kuwait. https://www.e.gov.kw/sites/kgoenglish/Pages/Visitors/AboutKuwait/KuwaitAtaGlaneGeographicalLocation.aspx.

[2] Kuwait Government Online Citizens and Residents. https://www.e.gov.kw/sites/kgoenglish/Pages/CitizensResidents/citizensAndResidents.aspx.

[3] Al-Awadi S.A., Moussa M.A., Naghuib K.K., Farag T.I., Teebi A.S., El-Khalifa M., El-Dossary L. (2008). Consanguinity among the Kuwaiti population. Clin. Genet..

[4] Tadmouri G.O., Nair P., Obeid T., Al Ali M.T., Al Khaja N., Hamamy H.A. (2009). Consanguinity and reproductive health among Arabs. Reprod. Health.

[5] Hoss G.R.W., Sperb-Ludwig F., Schwartz I.V.D., Blom H.J. (2020). Classical homocystinuria: A common inborn error of metabolism? An epidemiological study based on genetic databases. Mol. Genet. Genom. Med..

[6] El-Said M.F., Badii R., Bessisso M., Shahbek N., El-Ali M.G., El-Marikhie M., El-Zyoid M., Salem M., Bener A., Hoffmann G.F. (2006). A common mutation in theCBSgene explains a high incidence of homocystinuria in the Qatari population. Hum. Mutat..

[7] Gan-Schreier H., Kebbewar M., Fang-Hoffmann J., Wilrich J., Abdoh G., Ben-Omran T., Shahbek N., Bener A., Al Rifai H., Al Khal A.L. (2010). Newborn Population Screening for Classic Homocystinuria by Determination of Total Homocysteine from Guthrie Cards. J. Pediatr..

[8] Ismail H.M., Krishnamoorthy N., Al-Dewik N., Zayed H., Mohamed N.A., Di Giacomo V., Gupta S., Häberle J., Thöny B., Blom H.J. (2019). In silico and in vivo models for Qatari-specific classical homocystinuria as basis for development of novel therapies. Hum. Mutat..

[9] Refsum H., Fredriksen Å., Meyer K., Ueland P.M., Kase B.F. (2004). Birth prevalence of homocystinuria. J. Pediatr..

[10] Skovby F., Gaustadnes M., Mudd S.H. (2010). A revisit to the natural history of homocystinuria due to cystathionine β-synthase deficiency. Mol. Genet. Metab..

[11] Mudd S.H., Finkelstein J.D., Irreverre F., Laster L. (1964). Homocystinuria: An Enzymatic Defect. Science.

[12] Mudd S.H., Skovby F., Levy H.L. (1985). The natural history of homocystinura due to cystathionine β-synthase deficiency. Am. J. Hum. Genet..

[13] Morris A.A.M., Kožich V., Santra S., Andria G., Ben-Omran T.I.M., Chakrapani A.B., Crushell E., Henderson M.J., Hochuli M., Huemer M. (2017). Guidelines for the diagnosis and management of cystathionine beta-synthase deficiency. J. Inherit. Metab. Dis..

[14] Keller R., Chrastina P., Pavlikova M., Gouveia S., Ribes A., Kölker S., Blom H.J., Baumgartner M.R., Bártl J., Dionisi-Vici C. (2019). Newborn screening for homocystinurias: Recent recommendations versus current practice. J. Inherit. Metab. Dis..

[15] Huemer M., Kožich V., Rinaldo P., Baumgartner M.R., Merinero B., Pasquini E., Ribes A., Blom H. (2015). Newborn screening for homocystinurias and methylation disorders: Systematic review and proposed guidelines. J. Inherit. Metab. Dis..

[16] Al-Sadeq D.W., Nasrallah G.K. (2020). The Spectrum of Mutations of Homocystinuria in the MENA Region. Genes.

[17] ACT Sheets and Algorithms. https://www.acmg.net/ACMG/Medical-Genetics-Practice-Resources/ACT_Sheets_and_Algorithms.aspx.

[18] Wilcken B., Wiley V., Hammond J., Carpenter K. (2003). Screening Newborns for Inborn Errors of Metabolism by Tandem Mass Spectrometry. N. Engl. J. Med..

[19] Schulze A., Lindner M., Kohlmüller D., Olgemöller K., Mayatepek E., Hoffmann G.F. (2003). Expanded Newborn Screening for Inborn Errors of Metabolism by Electrospray Ionization-Tandem Mass Spectrometry: Results, Outcome, and Implications. Pediatrics.

[20] Rashed M.S., Ozand P.T., Bucknall M., Little D. (1995). Diagnosis of Inborn Errors of Metabolism from Blood Spots by Acylcarnitines and Amino Acids Profiling Using Automated Electrospray Tandem Mass Spectrometry. Pediatr. Res..

[21] Schulze A., Kohlmueller D., Mayatepek E. (1999). Sensitivity of electrospray-tandem mass spectrometry using the phenylalanine/tyrosine-ratio for differential diagnosis of hyperphenylalaninemia in neonates. Clin. Chim. Acta.

[22] Pei J., Li X.-Y. (2000). Determination of underivatized amino acids by high-performance liquid chromatography and electrochemical detection at an amino acid oxidase immobilized CuPtCl 6 modified electrode. Anal. Bioanal. Chem..

[23] Shipchandler M.T., Moore E.G. (1995). Rapid, fully automated measurement of plasma homocyst(e)ine with the Abbott IMx analyzer. Clin. Chem..

[24] Nexo E., Engbaek F., Ueland P.M., Westby C., O’Gorman P., Johnston C., Kase B.F., Guttormsen A.B., Alfheim I., McPartlin J. (2000). Evaluation of Novel Assays in Clinical Chemistry: Quantification of Plasma Total Homocysteine. Clin. Chem..

[25] Bártl J., Chrastina P., Krijt J., Hodík J., Pešková K., Kožich V. (2014). Simultaneous determination of cystathionine, total homocysteine, and methionine in dried blood spots by liquid chromatography/tandem mass spectrometry and its utility for the management of patients with homocystinuria. Clin. Chim. Acta.

[26] Stabler S.P., Korson M., Jethva R., Allen R.H., Kraus J.P., Spector E.B., Wagner C., Mudd S.H. (2013). Metabolic profiling of total homocysteine and related compounds in hyperho-mocysteinemia: Utility and limitations in diagnosing the cause of puzzling thrombophilia in a family. JIMD Reports.

[27] Mudd S.H. (2011). Hypermethioninemias of genetic and non-genetic origin: A review. Am. J. Med. Genet. Part C Semin. Med. Genet..

[28] Chace D.H., Hillman S.L., Millington D.S., Kahler S.G., Adam B.W., Levy H.L. (1996). Rapid diagnosis of homocystinuria and other hypermethioninemias from newborns’ blood spots by tandem mass spectrometry. Clin. Chem..

[29] Okun J.G., Gan-Schreier H., Ben-Omran T., Schmidt K.V., Fang-Hoffmann J., Gramer G., Abdoh G., Shahbeck N., Al Rifai H., Al Khal A.L. (2016). Newborn Screening for Vitamin B6 Non-responsive Classical Homo-cystinuria: Systematical Evaluation of a Two-Tier Strategy. JIMD Reports.

[30] Hoedt A.E.T., Van Kempen A., Boelen A., Duran M., Kemper-Proper E.A., Oey-Spauwen M.J.W., Wijburg F.A., Bosch A.M. (2007). High incidence of hypermethioninaemia in a single neonatal intensive care unit detected by a newly introduced neonatal screening programme. J. Inherit. Metab. Dis..

[31] Peterschmitt M.J., Simmons J.R., Levy H.L. (1999). Reduction of False Negati.ve Results in Screening of Newborns for Homocystinuria. N. Engl. J. Med..

[32] Turgeon C.T., Magera M.J., Cuthbert C.D., Loken P.R., Gavrilov D.K., Tortorelli S., Raymond K.M., Oglesbee D., Rinaldo P., Matern D. (2010). Determination of Total Homocysteine, Methylmalonic Acid, and 2-Methylcitric Acid in Dried Blood Spots by Tandem Mass Spectrometry. Clin. Chem..

[33] Naughten E.R., Yap S., Mayne P.D. (1998). Newborn screening for homocystinuria: Irish and world experience. Eur. J. Nucl. Med. Mol. Imaging.

[34] Sacharow S.J., Picker J.D., Levy H.L., Adam M.P., Ardinger H.H., Pagon R.A., Wallace S.E., Bean L.J.H., Mirzaa G., Amemiya A. (2004). Homocystinuria Caused by Cystathionine Beta-Synthase Deficiency. Reviews in GeneReviews® [Internet].

[35] Yap S., Naughten E. (1998). Homocystinuria due to cystathionine β-synthase deficiency in Ireland: 25 years’ experience of a newborn screened and treated population with reference to clinical outcome and biochemical control. J. Inherit. Metab. Dis..

[36] Bowron A., Barton A., Scott J., Stansbie D. (2005). Blood Spot Homocysteine: A Feasibility and Stability Study. Clin. Chem..

[37] Andersson A., Isaksson A., Hultberg B. (1992). Homocysteine Export from Erythrocytes and Its Implication for Plasma Sampling. Clin. Chem..

[38] Fiskerstrand T., Refsum H., Kvalheim G., Ueland P.M. (1993). Homocysteine and other thiols in plasma and urine: Automated determination and sample stability. Clin. Chem..

[39] Gramer G., Abdoh G., Ben-Omran T., Shahbeck N., Ali R., Mahmoud L., Fang-Hoffmann J., Hoffmann G.F., Al Rifai H., Okun J.G. (2017). Newborn screening for remethylation disorders and vitamin B12 deficiency-evaluation of new strategies in cohorts from Qatar and Germany. World J. Pediatr..

[40] Al-Dewik N., Ali A., Mahmoud Y., Shahbeck N., Ali R., Mahmoud L., Al-Mureikhi M., Al-Mesaifri F., Musa S., El-Akouri K. (2019). Natural history, with clinical, biochemical, and molecular characterization of classical homocystinuria in the Qatari population. J. Inherit. Metab. Dis..

[41] Yamada K., Yokoyama K., Aoki K., Taketani T., Yamaguchi S. (2020). Long-Term Outcomes of Adult Patients with Homocystinuria before and after Newborn Screening. Int. J. Neonatal Screen..

[42] Moammar H., Cheriyan G., Mathew R., Al-Sannaa N. (2010). Incidence and patterns of inborn errors of metabolism in the Eastern Province of Saudi Arabia, 1983–2008. Ann. Saudi Med..

[43] Al-Arrayed S., Hamamy H. (2011). The changing profile of consanguinity rates in bahrain, 1990–2009. J. Biosoc. Sci..

[44] Golbahar J., Al-Jishi E., Altayab D., Carreon E., Bakhiet M., Alkhayyat H. (2013). Selective newborn screening of inborn errors of amino acids, organic acids and fatty acids metabolism in the Kingdom of Bahrain. Mol. Genet. Metab..

[45] Wasim M., Awan F.R., Khan H.N., Tawab A., Iqbal M., Ayesha H. (2018). Aminoacidopathies: Prevalence, Etiology, Screening, and Treatment Options. Biochem. Genet..

[46] Al-Hammadi M.I. (2018). Presentation of Qatari Identity at National Museum of Qatar: Between Imagination and Reality. J. Conserv. Mus. Stud..

[47] Shibata N., Hasegawa Y., Yamada K., Kobayashi H., Purevsuren J., Yang Y., Dung V.C., Khanh N.N., Verma I.C., Bijarnia-Mahay S. (2018). Diversity in the incidence and spectrum of organic acidemias, fatty acid oxidation disorders, and amino acid disorders in Asian countries: Selective screening vs. expanded newborn screening. Mol. Genet. Metab. Rep..

[48] Zaidi S., Faiyaz-Ul-Haque M., Shuaib T., Balobaid A., Rahbeeni Z., Abalkhail H., Al-Abdullatif A., Al-Hassnan Z., Peltekova I., Al-Owain M. (2011). Clinical and molecular findings of 13 families from Saudi Arabia and a family from Sudan with homocystinuria. Clin. Genet..

[49] Silao C.L.T., Fabella T.D.F., Rama K.I.D., Estrada S.C. (2015). Novel cystathionine β-synthase gene mutations in a Filipino patient with classic homocystinuria. Pediatr. Int..

[50] Kaur R., Attri S.V., Saini A.G., Sankhyan N., Singh S., Faruq M., Ramprasad V.L., Sharda S., Murugan S. (2020). Seven novel genetic variants in a North Indian cohort with classical homocystinuria. Sci. Rep..

